# Microbial profiling of community-acquired pneumonia in patients with and without chronic obstructive pulmonary disease: a comprehensive molecular diagnostics study

**DOI:** 10.1186/s41479-025-00172-0

**Published:** 2025-08-05

**Authors:** Dagfinn Lunde Markussen, Christoffer Lindemann, Sondre Serigstad, Synne Jenum, Christian Ritz, Harleen M. S. Grewal

**Affiliations:** 1 Department of Clinical Science, Faculty of Medicine, Bergen Integrated Diagnostic Stewardship Cluster, University of Bergen, Bergen, 5020 Norway; 2 Department of Emergency Medicine, Haukeland University Hospital, Bergen, 5021 Norway; 3 Department of Microbiology, Haukeland University Hospital, Bergen, 5021 Norway; 4 Department of Infectious Diseases, Oslo University Hospital, Oslo, Norway; 5 National Institute of Public Health, University of Southern Denmark, Copenhagen, Denmark

## Abstract

**Background:**

Community-acquired pneumonia (CAP) causes substantial morbidity and mortality, particularly in patients with chronic obstructive pulmonary disease (COPD). This study compares the microbial detections in CAP patients with and without COPD using culture based and molecular diagnostic methods.

**Methods:**

This prospective study included 412 hospitalized pneumonia patients (136 with COPD). Lower respiratory tract samples were analysed with traditional cultures and a multiplex PCR panel (FilmArray Pneumonia Panel *Plus*). Multivariable Poisson regression identified predictors of *Pseudomonas aeruginosa* detection, and logistic regression estimated detection probability using the top predictors.

**Results:**

Overall pathogen detection rates were similar between groups, but *P. aeruginosa* was significantly more common in COPD patients (12.5% vs. 3.1%; *p* < 0.001). In adjusted analyses, each additional year of age increased the risk of *P. aeruginosa* by 5% (RR 1.05; 95% CI 1.01–1.09), while advanced COPD (GOLD 3–4) conferred a four‐fold higher risk (RR 4.29; 95% CI 1.94–9.46), diabetes mellitus a four‐fold risk (RR 4.04; 95% CI 1.97–8.29), and prior *P. aeruginosa* detection a five‐fold risk (RR 5.03; 95% CI 2.44–10.36). Inhaler use, bronchiectasis, and recent hospitalization were not independently associated.

**Conclusion:**

Although overall microbial detection rates were comparable between groups, *P. aeruginosa* was disproportionately prevalent in high-risk COPD individuals. While most COPD patients with pneumonia can be managed with standard empirical antibiotics, empirical coverage for *P. aeruginosa* should be considered for selected high-risk patients. Prospective studies are warranted to evaluate targeted *P. aeruginosa* coverage to optimize antibiotic stewardship and improve outcomes.

**Graphical Abstract:**

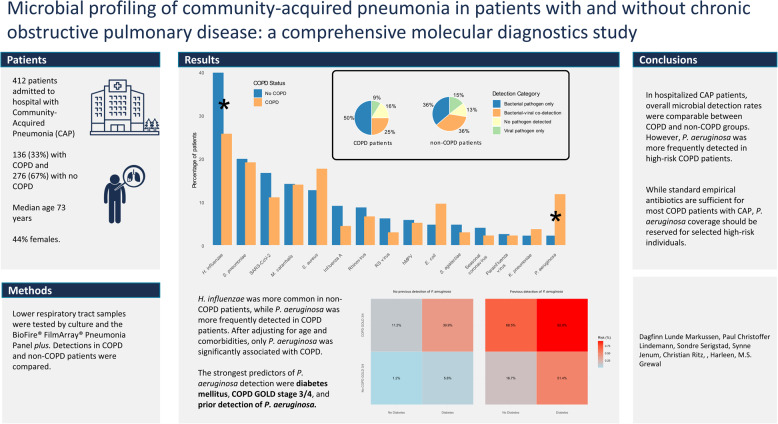

**Supplementary Information:**

The online version contains supplementary material available at 10.1186/s41479-025-00172-0.

## Introduction

Community-acquired pneumonia (CAP) remains a significant cause of morbidity and mortality worldwide, particularly among older adults and those with chronic respiratory conditions [1]. Chronic obstructive pulmonary disease (COPD) is a well-established risk factor for CAP, with COPD patients experiencing more frequent and severe pneumonia episodes compared to the general population [2–4]. However, the precise differences in microbial aetiology of CAP between COPD and non-COPD patients remain inadequately understood [5].

The lower respiratory tract microbiome in COPD patients is known to differ from that of healthy individuals, with reduced bacterial diversity and an increased abundance of specific pathogens potentially contributing to increased susceptibility to respiratory infections [5]. While previous studies have suggested that certain pathogens, such as *Haemophilus influenzae* and *Pseudomonas aeruginosa*, may be more prevalent in COPD-associated CAP, robust comparative data between COPD and non-COPD populations are limited [4, 6].

Accurate pathogen identification is crucial for optimizing CAP management [7]. While culture methods are the traditional gold standard, they suffer from limited sensitivity and slow turnaround times. Multiplex PCR panels, such as the FilmArray Pneumonia plus Panel, offer rapid and broad‐spectrum detection, potentially enhancing diagnostic yield [8].

Beyond COPD, various host factors such as advanced age, diabetes mellitus, bronchiectasis, colonization with resistant bacteria, and recent hospitalizations are known to shape the spectrum of pathogens involved in CAP [9]. These factors are often underexplored in studies focussing solely on COPD, despite their potential impact on patient outcomes and treatment decisions [4].

In this prospective study, we compared lower respiratory tract microbial profiles in hospitalized adults with CAP, stratified by COPD status using both traditional culture methods and a multiplex PCR panel (FilmArray Pneumonia plus Panel).We further evaluated how comorbidities influence pathogen distribution.

This research aims to fill a critical gap in the current literature by elucidating differences in pathogen prevalence and distribution between COPD and non-COPD patients with CAP. Such insights could have implications for tailoring diagnostic and therapeutic approaches in COPD versus non-COPD patients, ultimately improving outcomes in the management of CAP.

## Methods

### Study design and population

This investigation represents a post-hoc pooled analysis of three prospective studies: a feasibility study, a randomized controlled trial evaluating the impact of rapid molecular testing on antimicrobial therapy, and a subsequent cohort study [8, 10, 11]. All three studies used uniform inclusion and exclusion criteria and patients were included shortly after presentation in the emergency department (ED). The criteria have been detailed previously [8, 12]. Briefly, adults aged 18 and older who presented to the ED with suspected community-acquired pneumonia (CAP) and met at least two predefined clinical indicators were eligible for inclusion. Clinical indicators included symptoms such as new or intensified cough, expectoration, dyspnoea, haemoptysis, pleuritic chest pain, and fever (≥ 38.0⁰C), combined with radiological or auscultatory evidence of pneumonia. Exclusion criteria included recent hospitalization (within the last 14 days before ED presentation), cystic fibrosis, bronchiectasis requiring regular pulmonologist follow-up, palliative care status, or refusal to provide a lower respiratory tract (LRT) sample. Patients were enrolled between December 2, 2019, and April 30, 2023. A pause in inclusion from February to September 2020 occurred due to the COVID-19 pandemic. Although the individual cohorts were originally designed to explore different aspects of CAP diagnostics, the present study’s primary aim (comparing microbial detections in COPD versus non-COPD patients) was defined after data lock and was not a pre-specified endpoint of the original protocols.

For this analysis all patients with a discharge diagnosis of pneumonia who provided an LRT sample were included. Patients without an analysed LRT sample or without documentation of COPD status were excluded. COPD severity was categorized by GOLD stage using the most recent spirometry result documented in the medical record within the preceding 12 months. Only pathogens detected in at least 10 patients were considered in the analyses.

### Setting

The study was conducted at the Emergency Department of Haukeland University Hospital in Bergen, Norway, a teaching and primary healthcare institution serving approximately 430,000 residents and acting as a referral center for a regional population of about 1,000,000. During the study period, annual ED admissions ranged from 37,000 to 45,000.

### Data collection

Eligible patients were enrolled shortly after ED presentation. Baseline data were collected by study nurses or investigating physicians and recorded in the electronic case report form Viedoc (Viedoc Technologies, Uppsala, Sweden). Nasopharyngeal, oropharyngeal, or combined swabs were obtained from all patients for in-house PCR analysis to detect respiratory viruses and atypical pathogens.

Data on previous detections of *P. aeruginosa* was extracted from routine laboratory data using WHONET 2024 (whonet.org/, WHO Collaborating Centre for Surveillance of Antimicrobial Resistance for laboratory-based surveillance of infectious diseases and antimicrobial resistance). Test results preceding the admission date was included in the dataset.

### Sampling and microbiological testing

A concerted effort was made to obtain an LRT sample from each patient in the ED with the majority collected within 3 h of ED presentation and, whenever possible, prior to initiation of antibiotic therapy; any pre‐admission antibiotics were recorded. Nebulized isotonic or hypertonic saline (3% NaCl) was administered to facilitate sputum production in all patients. Preference was given to spontaneously expectorated purulent samples; if these were unavailable or of poor visual quality, sputum induction was used. In patients unable to expectorate despite induction—and with appropriate consent—endotracheal aspiration was performed. Study nurses and physicians trained in respiratory sampling oversaw the procedure to ensure consistency.Diagnostic assessments included standard-of-care methods: point-of-care PCR test (POCT) for SARS-CoV-2, LRT sample cultures, blood cultures, pneumococcal urine antigen tests, and in-house PCR for respiratory viruses and atypical bacteria. Additionally, LRT samples were analyzed using the BioFire FilmArray Pneumonia panel *plus* (FAP *plus*), a multiplex PCR panel detecting 27 bacterial and viral respiratory pathogens and seven antimicrobial resistance genes.

A direct head-to-head comparison of culture versus multiplex PCR was not performed in this analysis, but such comparisons for these cohorts have been reported previously [10, 11].

### Statistical analyses

#### Baseline characteristics

Baseline characteristics were summarized using means, medians, and proportions as appropriate for each variable. Summary statistics are presented for the overall population and for two subgroups: COPD patients versus non‐COPD patients. Comparisons of baseline characteristics between these groups were conducted using t-tests, chi-square tests, or Mann–Whitney U tests, depending on data distribution.

#### Imputations of missing data

Missing data were imputed using chained random forests with predictive mean matching [13]. This method was chosen due to its ability to address non-linear relationships and interactions among variables, improving the accuracy of imputations and reducing potential biases. To ensure imputed values were reasonable and unbiased, we compared the distribution of imputed and observed values. All baseline variables in the dataset were included in the imputation process, regardless of their use in the final analysis, to maximize information and accuracy. To avoid biasing key variables, the primary predictor (COPD status) and outcome variables (pathogen detections) were temporarily excluded from the dataset prior to imputation and reintroduced afterward.

#### Microbial detection analysis

To assess the association between COPD status and detection of individual microbial species, first fitted univariate Poisson regression models with robust (sandwich) standard errors to estimate crude risk ratios (RR). We elected Poisson regression over logistic regression because RR are more intuitive for clinicians and avoid the tendency of odds ratios to overestimate effects when outcomes are common, as is the case for several pathogens in our cohort (detection rates > 10%) [14, 15]. Microbial species yielding a univariate *p* < 0.1 were selected for multivariable analysis. For these species, we then fitted Poisson regression models using a generalized estimating equations (GEE) approach with an exchangeable correlation structure and robust standard errors to account for within‐patient clustering. Adjusted RR and 95% confidence intervals (CI) were reported for each model.Microbes that remained significantly associated with COPD after adjustment were further analysed to identify subgroups of COPD patients with higher detection rates. For this, an additional Poisson regression model was fitted, incorporating potential predictor variables identified from the literature and preceding analyses.

A significance level of 0.05 was used for all statistical tests. All analyses were performed using R software, version 4.4.1 (R Core Team (2024). R Foundation for Statistical Computing, Vienna, Austria. https://www.R-project.org/). The missRanger R package was used for the imputations [13].

## Results

**Fig. 1:**
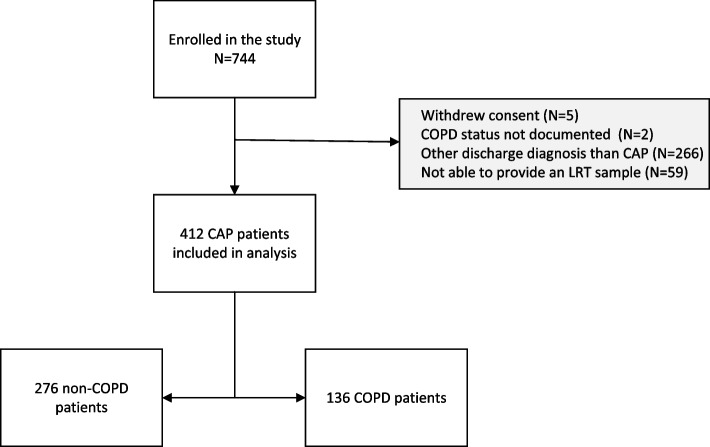
Study flowchart. Legend: The flowchart depicts the selection process for patients included in the study and reasons for exclusion. Abbreviations: COPD – Chronic Obstructive Pulmonary Disease. CAP – Community-Acquired Pneumonia. LRT – Lower Respiratory Tract

Of the 744 patients enrolled in the prospective studies, 412 CAP patients met the inclusion criteria for this analysis (Fig. 1). Among these, 136 (33%) had a pre-existing diagnosis of COPD, while 276 (67%) did not. Among the COPD patients, 33 patients (24%) had documented spirometry result consistent with GOLD stage 1 or 2, 62 (46%) with GOLD stage 3 or 4, and 41 (30%) lacked a recorded GOLD stage.

Significant differences were observed between COPD and non-COPD patients. COPD patients were older, had a higher burden of comorbidities, and presented with greater severity scores at admission compared to non-COPD patients (Table 1). Additionally, a larger proportion of COPD patients were current or former smokers.

**Table 1:** Baseline characteristics of included patients

Characteristic	Overall *n* = 412	Non-COPD Patients *n* = 276	COPD Patients *n* = 136	Difference (95% CI)^†^	*p*-value^‡^
Age, median (IQR)	73 (62, 80)	70 (53, 81)	75 (70, 80)	9 (6, 11)	< 0.001*
Sex, female, n (%)	181 (44%)	121 (44%)	60 (44%)	0.3 (−10, 11)	> 0.999
Co-morbidities					
Chronic respiratory failure	19 (5%)	7 (3%)	12 (9%)	6 (1, 12)	0.007*
Bronchiectasis	33 (8%)	21 (7%)	12 (9%)	2 (−5, 8)	0.745
Lung cancer	13 (3%)	7 (3%)	6 (4%)	2 (−3, 6)	0.433
Hypertension, n (%)	159 (39%)	113 (40%)	107 (39%)	−1 (−11, 10)	> 0.999
Coronary artery disease, n (%)	83 (20%)	40 (14%)	43 (32%)	17 (8, 27)	< 0.001*
Heart failure, n (%)	70 (17%)	32 (11%)	38 (28%)	17 (8, 26)	< 0.001*
CKD, n (%)	42 (10%)	27 (10%)	15 (11%)	1 (−7, 6)	0.826
Diabetes, n (%)	40 (10%)	25 (9%)	15 (11%)	2 (−5, 9)	0.646
Cancer, n (%)	34 (8%)	22 (8%)	12 (9%)	1 (−5, 7)	0.891
Immune deficiency, n (%)	42 (10%)	35 (13%)	7 (5%)	−7 (−13, −2)	0.028*
CCI score, median (IQR)	4 (1, 5)	3 (1, 4)	5 (4, 6)	2.1 (1.6, 2.5)	< 0.001*
Risk factors					
Current smoker, n (%)	62 (15%)	30 (11%)	32 (24%)	13 (4, 21)	< 0.001*
Pack years, median (IQR)	13 (0, 40)	3 (0, 25)	40 (11, 50)	37.5 (28, 40)	< 0.001*
Hospital admission last year, n (%)	181 (44%)	102 (37%)	79 (58%)	21 (11, 32)	< 0.001*
Antibiotic treatment past month, n (%)	151 (37%)	97 (35%)	54 (40%)	5 (−6, 15)	0.549
Severety scores at admission					
NEWS score, median (IQR)	5 (3, 6)	4 (2, 5)	6 (4, 7)	1.9 (1.5, 2.4)	< 0.001*
SOFA score, median (IQR)	2 (1, 3)	2 (1, 2)	2 (2, 3)	0.5 (0.3, 0.8)	< 0.001*
PSI score, median (IQR)	87 (66, 109)	80 (57, 105)	96 (78, 117)	18 (11, 24)	< 0.001*
CURB-65 score, median (IQR)	1 (1, 2)	1 (0, 2)	2 (1, 2)	0.7 (0.4, 0.9)	< 0.001*

*Abbreviations*: *COPD* Chronic Obstructive Pulmonary Disease, *CI* Confidence Interval, *IQR* Inter Quartile Range, *CKD* Chronic Kidney Disease, *CCI* Charlson Comorbidity Index, *NEWS* National Early Warning Score, *SOFA* Sequential Organ Failure Assessment, *PSI* Pneumonia Severity Index for Community-Acquired Pneumonia

^†^Percentage points difference with confidence interval for proportions and differences in medians for continous variables

^‡^*p*-values calculated using chi-squared test for dichotomous variables and Wilcoxon rank-sum test for continuous variables

^*^Significant

**Fig. 2:**
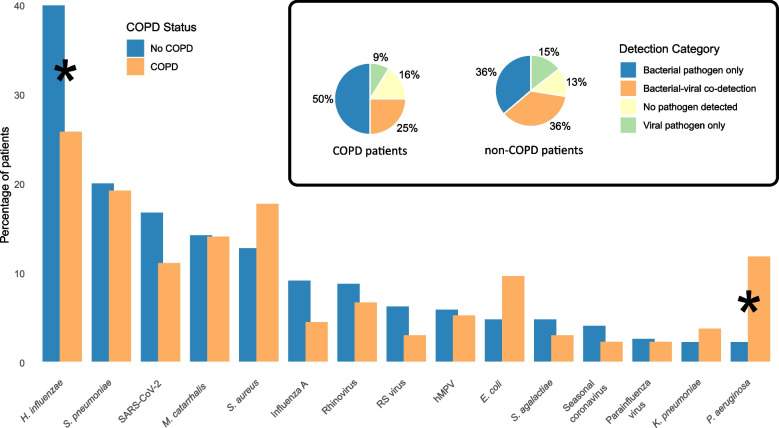
Detection rates of pathogens in COPD and non-COPD patients. Legend: The bar chart shows the percentage of patients with detected pathogens, with blue bars representing non-COPD patients and orange bars representing COPD patients. Asterisks (*) indicate statistically significant differences (*p* < 0.05) between the two groups. Statistical significance was determined using Fisher's exact test or chi-square test, depending on the expected cell counts. The pie charts illustrate the distribution of detection categories among COPD (left) and non-COPD (right) patients. Abbreviations: COPD—Chronic Obstructive Pulmonary Disease. SARS-CoV-2 – Severe Acute Respiratory Syndrome Coronavirus 2. RS virus—Respiratory syncytial virus. hMPV—Human metapneumovirus

In univariate analyses, *P. aeruginosa* was significantly more prevalent in COPD patients compared to non‐COPD patients (RR = 5.4, 95% CI: 2.2 to 13.5), whereas *H. influenzae* was more commonly detected in non‐COPD patients (RR for COPD: 0.7, 95% CI: 0.5 to 0.9). Detection rates of other bacteria or respiratory viruses did not differ significantly between the groups (Table 2, Fig. 2, Supplementary Table S1). Additionally, *E. coli* tended to be more frequently detected in COPD patients (RR = 2.0, 95% CI: 1.0 to 4.3; *p* = 0.06) and was therefore included in the multivariate analyses, one analysis per pathogen (*P.aeruginosa, H.influenzae, E.coli*), that corrected for age and comorbidities (mCCI).

**Table 2:** Detections

Characteristic	Overall *n* = 412 n (%)	Non-COPD Patients *n* = 276 n (%)	COPD Patients *n* = 136 n (%)	Relative Risk (95% CI)^†^	*p*-value^‡^
Viruses					
SARS-CoV-2	61 (15)	46 (17)	15 (11)	0.66 (0.38–1.14)	0.138
Rhinovirus	33 (8.0)	24 (8.7)	9 (6.6)	0.76 (0.36–1.59)	0.468
Respiratory Syncytial virus	21 (5.1)	17 (6.2)	4 (2.9)	0.48 (0.16–1.39)	0.176
Human Metapneumovirus	23 (5.6)	16 (5.8)	7 (5.1)	0.89 (0.37–2.11)	0.787
Influenza virus	31 (7.5)	25 (9.1)	6 (4.4)	0.49 (0.2–1.16)	0.104
Parainfluenza virus	10 (2.4)	7 (2.5)	3 (2.2)	0.87 (0.23–3.31)	0.838
Seasonal coronavirus	14 (3.4)	11 (4.0)	3 (2.2)	0.55 (0.16–1.95)	0.357
Bacteria					
* Streptococcus pneumoniae*	81 (20)	55 (20)	26 (19)	0.96 (0.63–1.46)	0.846
* Haemophilus influenzae*	145 (35)	110 (40)	35 (26)	0.65 (0.47–0.89)	0.007*
* Moraxella catarrhalis*	58 (14)	39 (14)	19 (14)	0.99 (0.59–1.64)	0.965
* Staphylococcus aureus*	59 (14)	35 (13)	24 (18)	1.39 (0.86–2.24)	0.175
* Escherichia coli*	26 (6.3)	13 (4.7)	13 (9.6)	2.03 (0.97–4.26)	0.061
* Pseudomonas aeruginosa*	22 (5.3)	6 (2.2)	16 (12)	5.41 (2.17–13.52)	< 0.001*
* Klebsiella pneumoniae*	11 (2.7)	6 (2.2)	5 (3.7)	1.69 (0.53–5.44)	0.378
* Streptococcus agalactiae*	17 (4.1)	13 (4.7)	4 (2.9)	0.62 (0.21–1.88)	0.402

*Abbreviations*: *COPD* Chronic Obstructive Pulmonary Disease, *CI* Confidence Interval

^†^Relative Risks (RR) and 95% Confidence Intervals (CI) were estimated using univariate GEE models with a Poisson distribution and a log link

^‡^*p*-values were calculated using the Wald test for the significance of the COPD variable in each model

^*^Significant

In these multivariate analyses, COPD remained strongly associated with the detection of *P. aeruginosa* (RR = 4.8, 95% CI: 1.9–12.3), whereas adjusting for age and the mCCI, no significant associations were found between COPD and the detection of *E. coli* or *H. influenzae*. Detection of *E. coli* was significantly associated with increasing age, with a risk ratio of 1.03 (95% CI: 1.00–1.08) per additional year. Conversely, the detection of *H. influenzae* was inversely associated with mCCI, with a risk ratio of 0.91 (95% CI: 0.85–0.98) per one-point increase in the score. Detailed results are presented in Table 3.

**Table 3:** Poisson regression models of associations between COPD status and pathogen detection adjusted for age, comorbidities, and CURB-65 score

Characteristic	*H. influenzae*		*E. coli*		*P. aeruginosa*	
	RR for detection (95% CI)	*p*-value	RR for detection (95% CI)	*p*-value	RR for detection (95% CI)	*p*-value
COPD	0.76 (0.55, 1.06)	0.109	1.69 (0.80, 3.56)	0.130	4.83 (1.90, 12.29)	0.001*
Age (years)	1.00 (0.99, 1.01)	0.930	1.03 (1.00, 1.08)	0.045*	1.05 (1.00, 1.10)	0.071
mCCI score	0.91 (0.85, 0.98)	0.017*	1.06 (0.92, 1.21)	0.473	0.94 (0.79, 1.13)	0.522

Abbreviations: *COPD* Chronic Obstructive Pulmonary Disease, *RR* Relative Risk, *CI* Confidence Interval, *mCCI* modified Charlson Comorbidity Index (excluding any points for chronic pulmonary disease)

### Analyses of risk factors for detection of *Pseudomonas aeruginosa*

As *P. aeruginosa* was the only microbe independently associated with COPD, we further investigated factors influencing its detection, both thorough univariate and multivariate analyses. A Poisson regression model was developed, incorporating key variables including age, COPD GOLD stage 3–4, mCCI score, diabetes mellitus, inhaler use, bronchiectasis, prior *P. aeruginosa* detection, and recent hospital admission. To enhance clinical interpretability, we then fitted a logistic regression model focusing on the three strongest predictors—COPD GOLD stage 3–4, diabetes mellitus, and prior *P. aeruginosa* detection—to estimate the probability of *P. aeruginosa* detection based on these key factors.

In the univariate analyses, patients with *P. aeruginosa* detection were more likely to have COPD (73% vs. 31%, *p* < 0.001), particularly GOLD stage 3–4 (55% vs. 13%, *p* < 0.001), and bronchiectasis (27% vs. 6.2%, *p* = 0.003). They were older (median age 77 vs. 72 years, *p* = 0.014) and more likely to use inhalators (86% vs. 38%, *p* < 0.001) and oral steroids (36% vs. 14%, *p* = 0.010). Additionally, these patients had higher Charlson Comorbidity Index scores (median 4 vs. 3, *p* = 0.009), more hospitalizations in the past year (82% vs. 42%, *p* < 0.001), and higher rates of recent nursing home admission (32% vs. 10%, *p* = 0.006). Detailed results are presented in Supplementary table S2.

**Fig. 3:**
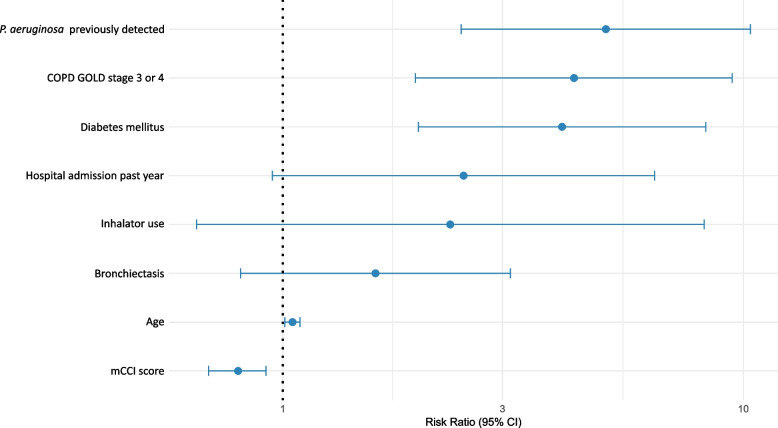
Forest plot of relative risks for *Pseudomonas aeruginosa* detection. Legend: This forest plot displays the relative risks (RRs) for the detection of *Pseudomonas aeruginosa* in patients with community-acquired pneumonia. RRs were estimated using a Poisson regression model. For categorical variables (e.g., COPD GOLD stage 3 or 4, diabetes mellitus, inhalator use, bronchiectasis, hospital admission in the past year, and previous detection of *Pseudomonas aeruginosa*), the RR represents the relative risk of detection when the variable is present compared to absent. For continuous variables (age and mCCI score), the RR represents the effect of a one-unit increase (one year for age, one point for mCCI score). The horizontal lines represent 95% confidence intervals for each RR, and the vertical line at RR = 1 denotes no association. Points to the right of this line indicate an increased risk, while points to the left indicate a reduced risk. Abbreviations: COPD – Chronic Obstructive Pulmonary Disease. GOLD – Global Initiative for Chronic Obstructive Lung Disease. mCCI – Modified Charlson Comorbidity Index (excluding points for pulmonary disease)

In the Poisson regression model, significant predictors for *P. aeruginosa* detection included age (RR = 1.05, 95% CI: 1.01–1.09), COPD GOLD stage 3–4 (RR = 4.29, 95% CI: 1.94–9.46, *p* < 0.001), diabetes mellitus (RR = 4.04, 95% CI: 1.97–8.29, *p* < 0.001), the modified Charlson Comorbidity Index (mCCI; RR = 0.80, 95% CI: 0.69–0.92, *p* = 0.002), and previous detection of *P. aeruginosa* (RR = 5.03, 95% CI: 2.44–10.36, *p* < 0.001). Use of inhalers, the presence of bronchiectasis, and hospital admission within the past year were not significantly associated with the detection of *P. aeruginosa*. Full results in Supplementary Table S3. These findings, including the relative risks and 95% confidence intervals, are illustrated in Fig. 3.

**Fig. 4:**
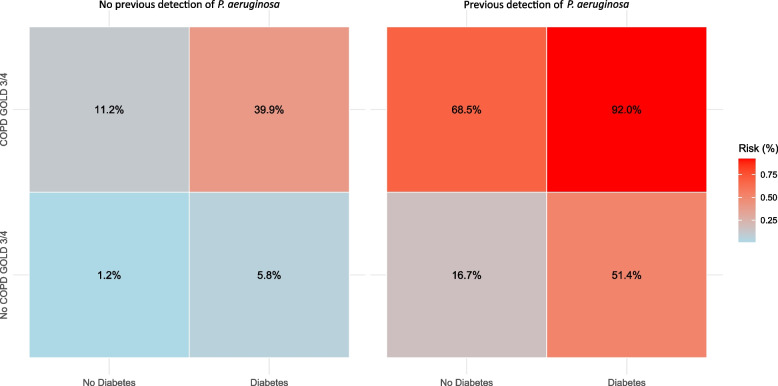
Heatmap of predicted risk for *Pseudomonas aeruginosa* detection. This heatmap illustrates the predicted probabilities of *Pseudomonas aeruginosa* detection for different combinations of three predictors: prior detection of *Pseudomonas aeruginosa*, presence of COPD GOLD stage 3 or 4, and diabetes mellitus. The probabilities were calculated using a logistic regression model with a logit link function. The model included the three predictors as independent variables. Each cell in the heatmap represents the predicted probability (converted from log-odds) for a unique combination of these predictors. The color gradient reflects the risk, ranging from low (blue) to high (red). Numeric values within each cell indicate the exact predicted probability of detection as a percentage. Abbreviations: COPD – Chronic Obstructive Pulmonary Disease. GOLD – Global Initiative for Chronic Obstructive Lung Disease

In the logistic regression model, the probability of *P. aeruginosa* detection was calculated using the three strongest predictors: COPD GOLD stage 3–4, diabetes mellitus, and previous detection of *P. aeruginosa* (all binary variables). When none of these predictors were present, the probability of detecting *P. aeruginosa* was 1.2%. In contrast, the probability increased dramatically to 92.0%. when all three were present. Figure 4 illustrates the probabilities associated with all possible combinations of these predictors, providing a comprehensive overview of risks based on their presence or absence.

## Discussion

This study compared lower respiratory tract detections in CAP patients with and without COPD, using both culture and the FilmArray Pneumonia *Plus* Panel. While Haemophilus influenzae was most common overall and similar between groups after multivariable adjustment, only *Pseudomonas aeruginosa* remained significantly associated with COPD status Severe COPD (GOLD stage 3–4), diabetes mellitus, older age, and prior detection of *P. aeruginosa* emerged as the principal predictors, with estimated prevalence rising from approximately 1% in low-risk patients to > 90% when all three factors co-occurred. In contrast, factors such as inhaler use, bronchiectasis, and recent hospital admissions did not significantly predict *P. aeruginosa* detection. These findings suggest that while most COPD patients with CAP can be effectively treated with standard empirical antibiotic regimens similar to non-COPD patients, consideration of *P. aeruginosa* coverage in high-risk individuals should be tailored to local *P. aeruginosa* prevalence and resistance patterns. Clinicians are advised to interpret our results in the context of regional microbiology data and stewardship guidelines to balance effective therapy against the risk of promoting resistance.

Our findings that microbial detections in CAP do not differ significantly between COPD and non-COPD patients align with a previous study from the German CAPNETZ Network. However, in contrast to our results, the CAPNETZ study found *H. influenzae* to be significantly more frequent in COPD patients [4]. Notably, the CAPNETZ study only reported crude detection rates without adjusting for confounders. Compared to the CAPNETZ study, where microbiologic diagnostics were left to routine local practices, our study has the advantage of using a standardized diagnostic package that included a concerted effort to obtain LRT samples and employed a syndromic multiplex PCR panel. The CAPNETZ study did not find a significant difference in *P. aeruginosa* detections, with only seven detections in 1,288 patients. In line with our results, a large Spanish cohort also reported increased *P. aeruginosa* prevalence in COPD-associated CAP [16].

Our finding that *P. aeruginosa* is significantly more prevalent in COPD patients with CAP aligns with the multinational point-prevalence study by Restrepo et al., which enrolled 3,193 hospitalized CAP patients across 54 countries and reported an overall *P. aeruginosa* prevalence of 4.2%, rising to 67% among those with prior colonization and at least one severe chronic lung disease (GOLD stage 3–4, bronchiectasis, or tracheostomy) [17]. Interestingly, while some studies have suggested that bronchiectasis and frequent use of inhalers may contribute to increased risk of *P. aeruginosa* infections in COPD patients, our analysis did not find these factors to be significant predictors [16, 17]. This discrepancy may may stem from our prospective design, stringent lower‐respiratory sampling, and the evolution of COPD management guidelines since earlier cohorts [16, 18].

The association between diabetes mellitus and *P. aeruginosa* detection in CAP patients is also consistent with existing literature. Diabetes is known to impair immune function, increasing the risk of various infections, including those caused by *P. aeruginosa* [19, 20]. Our study reinforces this relationship, highlighting diabetes as a significant predictor alongside severe COPD and prior *P. aeruginosa* detection.

This study has several limitations. First, our inclusion criteria required the ability and willingness to provide an LRT sample which may bias our cohort toward milder disease and underrepresent pathogens prevalent in critically ill patients [8]. Consequently, our findings are most applicable to hospitalized adults with mild to moderate CAP who can produce sputum. Second, the modest number of *P. aeruginosa* detections limits statistical power for subgroup analyses and may overestimate associations in high-risk strata. However, our results align with previous studies. Additionally, as a single-center study in Norway—a setting characterized by low baseline antibiotic resistance and consumption, our pathogen prevalence and resistance patterns may not reflect regions with higher resistance burdens or different antibiotic practices [21]. Additionally, the absence of numerical spirometric measures and standardized data on prior-year exacerbation frequency limits the precision of our COPD severity classification and may have influenced the observed associations with pathogen detection. It is also important to acknowledge a limitation common to all studies on the aetiology of CAP; the inability to distinguish between pathogens that cause infection and those that merely colonize the respiratory tract [10]. The assessment of specificity and positive predictive value is hindered by the absence of a definitive gold standard for pneumonia aetiology and by typically low pathogen recovery from normally sterile sites (e.g., blood, pleural fluid) [9, 22].

## Conclusion

In conclusion, our study demonstrates that the majority of COPD patients hospitalized with CAP can be effectively managed with standard empirical antibiotic regimens similar to those used for non-COPD patients. However, empirical antipseudomonal coverage may be considered in selected high-risk patients (advanced COPD, diabetes, prior *P. aeruginosa* detection), but should always be interpreted in light of local *P. aeruginosa* prevalence and resistance rates. It is important to recognize that the detection of *P. aeruginosa* in lower respiratory tract samples does not necessarily indicate it as the causative agent of infection. Moreover, previous studies have not demonstrated improved clinical outcomes when empirical treatment for *P. aeruginosa* was administered in COPD patients with CAP [23]. Therefore, future research should prospectively identify specific patient populations who would benefit most from empirical *P. aeruginosa* coverage. Such studies are essential to optimize antibiotic stewardship, enhance patient outcomes, and reduce the risks associated with use of unnecessary broad-spectrum antibiotics.

## Supplementary Information

Supplementary Material 1.

## Data Availability

The raw datasets generated and analysed during the current study are not publicly available due to privacy concerns under the European General Data Protection Regulation (GDPR). However, de-identified participant data and aggregated data presented in this study can be made available from the corresponding author upon reasonable request. Data sharing will be contingent on the receiving institution's agreement to a Data Use Agreement.

## Abbreviations

CCI: Charlson Comorbidity Index
CAP: Community-Acquired Pneumonia
CI: Confidence Interval
CKD: Chronic Kidney Disease
COPD: Chronic Obstructive Pulmonary Disease
FAP plus: BioFire FilmArray Pneumonia panel plus
GEE: Generalized Estimating Equations
GOLD: Global Initiative for Chronic Obstructive Lung Disease
hMPV: Human metapneumovirus
IQR: Inter Quartile Range
LRT: Lower Respiratory Tract
mCCI: Modified Charlson Comorbidity Index (excluding pulmonary disease)
NEWS: National Early Warning Score
POCT: Point-of-Care PCR Test
PSI: Pneumonia Severity Index for Community-Acquired Pneumonia
RR: Relative Risk
RS virus: Respiratory syncytial virus
SARS-CoV-2: Severe Acute Respiratory Syndrome Coronavirus 2
SOFA: Sequential Organ Failure Assessment
