# Supplementary material for: Microbial profiling of community-acquired pneumonia in patients with and without chronic obstructive pulmonary disease: a comprehensive molecular diagnostics study
Source: Pneumonia (Nathan). 2025 Aug 5;17:19. doi: 10.1186/s41479-025-00172-0 (PMC12323216; doi:10.1186/s41479-025-00172-0)
Supplement: Supplementary file 1 — Supplementary Material 1. [file 41479_2025_172_MOESM1_ESM.docx]

# Supplementary material

## Supplementary Table S1: Number of microbial detections in COPD and non-COPD patient

| Median number of detections | | | | | | | | | | |
| --- | --- | --- | --- | --- | --- | --- | --- | --- | --- | --- |
| **Detection Type** | **Median (COPD)** | **Q1 (COPD)** | **Q3 (COPD)** | **Median (No COPD)** | **Q1 (No COPD)** | **Q3 (No COPD)** | **Median Difference** | **CI Lower** | **CI Upper** | **P-Value^1^** |
| Total detections | 1.00 | 1.00 | 2.00 | 1.00 | 1.00 | 2.00 | 0.00 | −1.00 | 1.00 | 0.317 |
| Viral detections | 0.00 | 0.00 | 1.00 | 1.00 | 0.00 | 1.00 | −1.00 | −1.00 | 0.00 | 0.001* |
| Bacterial detections | 1.00 | 0.75 | 2.00 | 1.00 | 0.00 | 2.00 | 0.00 | 0.00 | 0.00 | 0.594 |

^1^Wilcoxon rank sum test

## Supplementary Table S2: Clinical characteristics in patient with and without a detection of *Pseudomonas aeruginosa*

| **Characteristic** | *P. aeruginosa* not detected**, N = 390***^1^* | *P. aeruginosa* detected**, N = 22***^1^* | **p-value***^2^* |
| --- | --- | --- | --- |
| **COPD** | 120 (31%) | 16 (73%) | <0.001 |
| **Age** | 72 (61, 80) | 77 (72, 85) | 0.014 |
| **Sex** |  |  | 0.9 |
| Female | 171 (44%) | 10 (45%) |  |
| Male | 219 (56%) | 12 (55%) |  |
| **Asthma** | 42 (11%) | 3 (14%) | 0.7 |
| **COPD GOLD 1-2** | 30 (7.7%) | 3 (14%) | 0.4 |
| **COPD GOLD 3-4** | 50 (13%) | 12 (55%) | <0.001 |
| **COPD unknown stage** | 40 (10%) | 1 (4.5%) | 0.7 |
| **Chronic respiratory failure** | 19 (4.9%) | 0 (0%) | 0.6 |
| **Lung cancer** | 13 (3.3%) | 0 (0%) | >0.9 |
| **Bronchiectasis** | 24 (6.2%) | 6 (27%) | 0.003 |
| **Current smoker** | 60 (15%) | 2 (9.1%) | 0.6 |
| **Pack years** | 11 (0, 40) | 25 (7, 39) | 0.4 |
| **Inhalator use** | 150 (38%) | 19 (86%) | <0.001 |
| **Chronic steroide use** | 54 (14%) | 8 (36%) | 0.010 |
| **Contact with children in daycare past 14 days** | 130 (34%) | 8 (38%) | 0.7 |
| **Hypertension** | 153 (39%) | 6 (27%) | 0.3 |
| **Heart failure** | 62 (16%) | 5 (23%) | 0.4 |
| **Atrial fibrillation** | 70 (18%) | 2 (9.1%) | 0.4 |
| **Peripheral vascular disease** | 19 (4.9%) | 1 (4.5%) | >0.9 |
| **Stroke** | 44 (11%) | 1 (4.5%) | 0.5 |
| **Chronic kidney disease** | 39 (10%) | 3 (14%) | 0.5 |
| **Diabetes mellitus** | 33 (8.5%) | 7 (32%) | 0.003 |
| **Liver failure** | 2 (0.5%) | 0 (0%) | >0.9 |
| **Cancer** | 33 (8.5%) | 1 (4.5%) | >0.9 |
| **Immune deficiency** | 40 (10%) | 2 (9.1%) | >0.9 |
| *^1^* Median (IQR); n (%) | | | |
| *^2^* Wilcoxon rank sum test; Pearson’s Chi-squared test; Fisher’s exact test | | | |

## Supplementary table S3: Poisson Regression Results. Relative Risks (RR) for detection of *P. aeruginosa* with 95% Confidence Intervals and p-values

| **Predictor** | **Risk Ratio** | **95% CI** | **P-Value** |
| --- | --- | --- | --- |
| Modified CCI score | 0.800 | 0.69 to 0.92 | 0.002* |
| Age (years) | 1.050 | 1.01 to 1.09 | 0.011* |
| Bronchiectasis | 1.590 | 0.81 to 3.12 | 0.173 |
| Inhalator use | 2.310 | 0.65 to 8.22 | 0.196 |
| Hospital admission past year | 2.470 | 0.95 to 6.42 | 0.063 |
| Diabetes mellitus | 4.040 | 1.97 to 8.29 | <0.001* |
| COPD stage 3 or 4 | 4.290 | 1.94 to 9.46 | <0.001* |
| *Pseudomonas aeruginosa* previously detected | 5.030 | 2.44 to 10.36 | <0.001* |
